# Relationship between salivary lactoferrin level and brain amyloid load in periodontal and non-periodontal subjects

**DOI:** 10.4317/medoral.27612

**Published:** 2025-10-17

**Authors:** José Antonio Gil-Montoya, María José Gerez-Muñoz, Eva M Triviño-Ibáñez, Eva Rosel, Manuel Bravo, Juan Carlos Rómero-Fábrega, Sonia Morales-Santana, Manuel Gómez-Río

**Affiliations:** 1 School of Dentistry. University of Granada. Paseo de Cartuja s/n, 18071, Granada, Spain.; 2 Granada Institute for Biomedic Research. Avenida de Madrid, 15, 18012, Granada, Spain.; 3 Department of Nuclear Medicine, University Hospital Virgen de las Nieves. Avenida de las Fuerzas Armadas, 2, 18014 Granada, Spain.; 4 Cognitive and Behavioral Neurology Unit, University Hospital Virgen de las Nieves. Avda. Juan Pablo II s/n, 18013, Granada, Spain.; 5 Proteomic Research Unit, Granada Institute for Biomedic Research, Avenida de Madrid, 15, 18012, Granada, Spain.

## Abstract

**Background:**

Lactoferrin in saliva has been proposed as a possible diagnostic biomarker for Alzheimer's disease, as it is associated with ß-amyloid load in the brain. The aim of this study was to find out whether there is an association between salivary lactoferrin and cerebral ß-amyloid load and the involvement of periodontal disease in this possible connection.

**Material and Methods:**

Six exploratory comparison groups were designed: participants with mild cognitive impairment (n=50) (positive PET-amyloid with and without periodontal disease and negative PET-amyloid with and without periodontal disease) and cognitively normal older individuals with and without periodontal disease (n=19). All participants were recruited from referral hospitals in Granada, Spain, and from a nursing home in the same socio-economic area as the hospital participants. A salivary lactoferrin determination and a periodontal assessment has been performed in each of the participants.

**Results:**

The results show that both having an atypical ß-amyloid load in the brain (PET+) and having periodontal disease are clearly associated with a lower concentration of salivary lactoferrin (p=0.011 and p=0.032), but not with age or gender.

**Conclusions:**

In this studio, the positive PET-amyloid and periodontal disease are related independently with lower lactoferrin levels.

## Introduction

Alzheimer's disease (AD) is considered to be the most prevalent dementia worldwide, with no causal hypothesis fully accepted. The etiology is complex and multifactorial, involving both genetic and environmental factors. Neuroinflammation, abnormal amyloid (ßA) load and tau hyperphosphorylation are considered clear hallmarks in the pathogenesis of AD (1). In turn, neuroinflammation appears to be triggered by various blood-brain barrier dysfunctions or by the action of certain peripheral infections, which hypothetically activate the production of ßA protein (2) after causing low-grade systemic inflammation.

Beside intestinal dysbiosis, one of the most studied peripheral infections as a possible etiological source of AD is periodontal disease (PD) (3). Specifically, the role of Porphyromonas gingivalis (Pg) has gained special interest in the scientific community in recent years, being considered the key stone pathogen of the PD (3). In addition to its hypothetical role in the activation of brain ßA protein in animal models (4), recent published articles suggest that Pg was found in AD-diagnosed brain autopsy specimens (5 , 6). The entry of these periodontopathogens into brain tissue may be through the trigeminal nerve, circumventricular organs, perivascular spaces and olfactory unsheathing cells acting as trojan horses (7), although it seems that the most accepted route is directly through the blood-brain barrier due to vascular endothelial barrier disruption and increased permeability destroying intercellular junctions (1). Once it reaches the brain, a probably aberrant brain immune response is triggered, resulting in neuroinflammation, abnormal ßA protein formation and eventual neuronal destruction (8 , 9). Given that periodontal disease is a modifiable risk factor, confirmation of its involvement in the etiology of AD would be a further element to consider in preventing the onset of AD.

Derived from the above hypothesis, multiple lines of evidence have emerged, such as the potential link between AD and antimicrobial protein like lactoferrin (Lf) (9 - 11). Recent reports demonstrating that decreased salivary levels of Lf are found as excellent early diagnostic performance markers to detect prodromal AD and AD dementia and actually distinguishing them from other dementia as frontotemporal dementia or non-amyloid dementias, with sensitivities and specificities over 87% and 91% respectively (9). It has also been shown that salivary Lf is reduced in early and late-onset AD compared with cognitive healthy subject supporting that it could be a technically reliable and useful biomarker in AD (11).

As a result of these findings, a positive feedback loop in the salivary lactoferrin-AD relationship has been proposed. Some authors suggest that the hypothalamic alteration associated with AD causes via cholinergic parasympathetic nerves an alteration in salivary secretion and consequently a decrease in proteins such as Lf. In turn, this decreases in Lf and thus in the antimicrobial capacity of saliva, may represent a disruption in the defensive line against pathogens, including Pg that would have more possibilities of proliferating (10 , 11). To confirm or reject this hypothesis, the present clinical study was designed to determine whether there is an association between salivary lactoferrin and AD and the involvement of periodontal disease in this association.

## Material and Methods

Design

An observational study with three variables or factors was performed. Those variables were cognitive impairment (MCI yes/no), amyloid PET (positive/negative) and periodontal disease (No/Yes). In the absence of accepted clinical values to declare a difference in LF as significant, we focus the sample size estimation according to Cohen's scale, which offers general useful rules derived from the standardized differences for the effects. Forty participants with MCI and twenty controls are needed to detect, with 80% power and 5% alpha error, significant differences in standardized Lf (log10) of 0.8, substantial according to Cohen's scale and according to Sample Power 2.0 (SPSS Inc., Chicago, IL).

Population

The study was finally designed with 69 participants, of whom 50 had suspected MCI and different degrees of periodontal disease and 19 cognitively healthy controls. The participants with MCI came from the Nuclear Medicine Service of the Hospital Virgen de las Nieves in Granada (HUVN), under study for cognitive impairment in filiation, in whom, in the opinion of the neurologists responsible for the dementia area, PET-amyloid scan is required as an imaging biomarker to confirm or rule out possible cognitive impairment with suspected AD. All participants were previously evaluated in the Neurology Service of the same hospital, from a clinical point of view by means of a guided anamnesis, general/neurological examination and detailed neuropsychological evaluation. The diagnostic criteria for this group were those commonly accepted in the field (the modified Petersen criteria (12) were used for MCI and the revised IWG-2/AAA criteria (13) were used for AD-related impairment).

The cognitively normal older individuals (reference group) were recruited from a nursing home in the same socio-economic area as the hospital participants. The cognitive assessment of these subjects was carried out by the medical staff of the center, performing the same examinations as in the participants with suspected MCI: Guided anamnesis, general/neurological examination and detailed neuropsychological assessment. For ethical reasons, these cognitively normal older individuals did not undergo amyloid PET scan.

The general inclusion criteria for participation in the study were to be under study for cognitive impairment in affiliation or to have a cognitive status in accordance with their age in the case of the reference group. All participants gave informed consent to the protocol approved by the Ethical Committee of Granada University, nº: 2684/CEIH/2022. Subjects with less than 6 teeth in the mouth, those who had received dental treatment in the last 3 months, those with metabolic disorders (hypothyroidism, B12 or folic acid deficiency), known psychiatric disease (schizophrenia or depression), cerebrovascular disease, neurological disease (Parkinsonian syndrome, epilepsy, etc) or participants with difficulty or impossibility to provide an unstimulated saliva sample or to receive an oral and periodontal examination were excluded.

PET acquisition

The brain ßA load have been assessed by Amyloid-PET/TC scan as previously described (14), following international consensus guidelines (15). In summary, imaging have been acquired 90-110 min after intravenous injection of 300 MBq of [18F]Florbetaben. After reconstruction, data set of axial imaging have been evaluated visually by specialist, classifying the exploration as positive [+] or negative (-) on basis radiopharmaceutical uptake in cortical standardized regions of interest (16 , 17).

Salivary lactoferrin analysis

Whole saliva was collected from participants with suspected MCI and from cognitively normal older individuals. All participants were required to fast for a minimum of 8 hours prior to saliva collection. Furthermore, they were instructed to abstain from drinking, eating, and smoking before the sampling process. The presence of blood contamination in the saliva samples was ruled out through visual inspection.

The collected saliva samples were placed in 1 mL sterile tubes, immediately placed on ice, and treated with a Protease Inhibitor Cocktail (Roche). Samples were then centrifuged at 1,500 rpm for 10 minutes at 4°C to remove any debris. Each 1 mL stock sample was then aliquoted into several containers of 0.1 mL. These aliquots were promptly stored at -80°C to prevent multiple freeze/thaw cycles and preserve sample integrity.

The levels of Lf in the saliva samples were quantified using the Lactoferrin Human ProcartaPlex Simplex Kit (EPX010-12250-901, Invitrogen), following the manufacturer's instructions. All analyses were performed on a Luminex 200 Workstation (Bio-Rad).

To determine the appropriate dilution factor, different sample dilutions of 1:100, 1:1,000, 1:10,000, and 1:100,000 were tested. A dilution factor of 1:10,000 was found to be optimal, with concentrations within the linear range of the standard curve. Each sample was assayed in duplicate, and the mean values from these duplicates were used as single data points. The data were expressed in pg/mL but were converted to log10 for statistical treatment.

Precision was evaluated by calculating intra-assay and inter-assay variations. The intra-assay coefficient of variation was less than 10%, and the inter-assay coefficient of variation was less than 15%. The assay range was 45.17-46,250 pg/mL. The limit of quantification was 0.1 pg/mL, determined using a sample concentration near the expected lower limit of detection.

Periodontal disease assessment

The oral and periodontal examinations as well as the collection of saliva samples were carried out by a single researcher (MJG). They were performed under artificial light conditions in the same place where the radiopharmaceutical was injected. The protocol proposed by the WHO for this activity was basically followed. All teeth presents were explored (with 6 exploration sites per tooth) except those teeth for which extraction was indicated due to severe destruction of the clinical crown and third molars. The maximum value per tooth for each periodontal variable measured (pocket depth, clinical attachment loss, bleeding on probing and plaque index) was recorded.

The definition of 'periodontal disease' recently agreed upon by the American Academy of Periodontology and the European Federation of Periodontology was used (18). A case of periodontitis was considered if interdental CAL is detectable at 2 non-adjacent teeth or buccal or oral CAL 3mm with pocketing &gt;3mm is detectable at 2 teeth. The observed CAL could not be attributed to non-periodontal causes such as gingival recession of traumatic origin, dental caries or distal aspect of the second molars due to the presence of the third molar.

Statistical analysis was performed with IBM SPSS Statistics 22.0 (IBM Corp., Armonk, NY), using the descriptive and analytical methods described at the bottom of each table.

## Results

A total of 69 subjects were explored, 50 of them with suspected mild cognitive impairment and 19 cognitively normal older individuals according to the neurological examination conducted. In the total sample the age range of the participants was 38-94 (mean±sd=70.3±11.9) and 47 of 59 were female. Table 1 presents these variables by group, with a higher age in the reference group. Among participants with MCI, 26 subjects had a positive amyloid PET (PET+) and 24 negatives. Considering the whole sample, 28 of 69 (40.5%) individuals presented PD.

Table 1

Table 2 shows, by groups, the description of lactoferrin and its Log10, since, due to its very asymmetric distribution, it will be used in the analytical statistics. That statistical analysis is performed in Table 3.

Table 2

Table 3 shows the univariate and multivariate effect, by means of multiple linear regression of the study variables on the log10 of lactoferrin. In the multivariate analysis, it is observed that compared to the reference group, both having an atypical ßA load in the brain (PET+) and having PD are clearly associated with a lower concentration of salivary lactoferrin (p=0.011 and p=0.032 respectively). Further analysis indicates, the interaction between the variable Group and PD is not significant (p=0.231.)

Table 3

The discriminant capacity of lactoferrin to differentiate cases (mild cognitive impairment) versus reference group is not significant (area under the ROC curve=0.60, p=0.189, results not shown in Tables). However, it is significant for differentiating PET+ cases versus PET- cases (Table 4.)

Table 4

## Discussion

This exploratory analysis of salivary Lf behavior in PET+ and PET- cognitively impaired participants, with or without PD, confirms previously published findings of lower salivary Lf detection in participants with higher brain amyloid load. Furthermore, it is observed that PD is also associated with a lower salivary Lf concentration independently of ßA load and that PET- and periodontally healthy participants had the highest salivary Lf concentration.

Lf is a salivary protein that has generated great interest in the scientific community in recent years because of its possible involvement in the infectious hypothesis of periodontal origin in AD. It is a protein with clear antimicrobial functions against oral pathogens and control of the oral microbiome (19) and with a clear involvement in the innate immune system, which makes it a candidate for explaining the infectious hypothesis of AD (20) or rather the innate immune system dysregulation hypothesis proposed by some authors(9). Several studies have reported decreased salivary lactoferrin levels in participants suffering from mild cognitive impairment and AD compared with age-matched healthy subjects (21), indicating a putative link between AD, the immune system, and brain infections (22). However, it is not well known how the main source of AD-associated oral pathogens, Pg, is associated with the presence of Lf in cases of cognitive impairment of amyloid origin.

In our study, besides corroborating the decrease in salivary Lf in participants with abnormal ßA load, we observed that in participants with PD, both in the group of PET+ and PET- participants, the level of salivary lactoferrin was lower than in the other groups. However, most studies on salivary Lf and PD show an increase in Lf in case of active PD and, in fact, it is proposed to be used together with other proteins as prognostic and diagnostic biomarkers of PD (23). In our opinion, this apparent contradiction could be explained by the modulatory role of the hypothalamus in salivary secretion, as proposed by Carro's group in their hypothesis (21). These researchers propose a positive feedback loop, where AD-related hypothalamic dysfunction may lead dysregulation of salivary gland resulting in decreased salivary lactoferrin secretion (10). Furthermore, it is unclear when Lf levels began to drop in participants suffering from AD (11), although the researchers conclude in their manuscript that the decrease in Lf levels is greater in participants with late-onset AD versus early-onset AD. Therefore, at the time of our study, in participants diagnosed with AD (PET+), the decrease in Lf production was already a fact. If we understand the presence of MCI as a previous step to the appearance of an amyloid disease such as AD (not in all cases), we may be facing an early diagnostic biomarker, supported by the fact that in our results, although it was only significant in PET+ cases, there is an association between participants with cognitive impairment and the decrease in Lf levels.

The results obtained should be interpreted with caution, given the exploratory factorial design of the study and the small sample size. Although the latest scientific evidence, discussed above, shows a clear association between Lf levels and ßA load, not all the studies conclude in the same direction, although the authors justify this by a more heterogeneous sample composition than in the studies where there was an association (24). Therefore, more follow-up studies with larger samples are needed to confirm these positive results. If so, this would be a possible biomarker, easy to use and with the possibility of using it in conjunction with a simple periodontal examination to detect avoidable oral pathology as an infectious source and consequently, take appropriate preventive periodontal measures.

## Conclusions

In this studio the positive amyloid-PET/TC and periodontal disease are related independently with lower Lf levels. Further studies are needed to confirm the role of Lf as an early diagnostic biomarker of Alzheimer's disease.

## Figures and Tables

**Table 1:** Table Gender and age of the sample.

	Cognitive Impairment PET+ n=26		Cognitive Impairment PET- n=24		Cognitive normal PET unknown n=19			
		
Variable	PD-	PD+	PD-	PD+	PD-	PD+	p-global	Paired comparison
	[A] (n=15)	[B] (n=11)	[C] (n=12)	[D] (n=12)	[E] (n=14)	[F] (n=5)		
Gender, n (%)							0.165a	
Women	12 (80.0)	8 (72.7)	7 (58.3)	5 (41.7)	15 (78.9)			
Men	3 (20.0)	3 (27.3)	5 (41.7)	7 (58.3)	4 (21.1)			
Age (years)							<0.001b	ABCD Ec
Range	51-84	59-81	57-70	38-74	75-94	76-91		
mean±sd	65±8	68±7	64±4	61±10	85±7	85±6		

aChi-square;bANOVA; cStudent-Newman-Keuls; "≠" means p<0.05.

**Table 2:** Table Lactoferrin. Description of study participants.

	Cognitive Impairment, PET+		Cognitive Impairment, PET-		Cognitive normal, PET unknown	
Variable	PD-	PD+	PD-	PD+	PD-	PD+
	[A] (n=15)	[B] (n=11)	[C] (n=12)	[D] (n=12)	[E] (n=14)	[F] (n=5)
Lactoferrin (pg/ml.)						
Range	0.14-2798	0.14-244	3.82-3528	0.01-734	25.2-897	1.6-275
mean±sd	304±753	56±86	646±1193	166±209	402±332	70±105
Lactoferrin (Log10)						
Range	-0.85-3.45	-0.85-2.39	0.58-3.55	-2.00-2.87	1.40-2.95	0.21-2.44
mean±sd	1.36±1.12	1.19±0.92	2.04±0.92	1.54±1.35	2.37±0.57	1.39±0.70

2

**Table 3:** Table Linear regressions with dependent variable Log10 of Lactoferrin (n=69).

	Univariate		Multivariatea	
Variable	βse	p-value	Î²±ee	p-value
Age (yrs.)	0.00±0.01	0.589	-	-
Gender (Female)	-0.16±0.25	0.530	-	-
Group				
Cognitive Impairment, PET+	-0.62±0.27	0.023	-0.64±0.25	0.011
Cognitive Impairment, PET-	-0.11±0.29	0.698	-0.10±0.27	0.710
Cognitive normal, PET unknown (Ref)	0.00±0.00		0.00±0.00	
Periodontal disease	-0.47±0.24	0.057	-0.51±0.23	0.032

aConstructed by forward stepwise procedure (p0.15 to exclude). The interaction GroupxPeriodontal disease is not significant (p=0.231).

**Table 4:** Table Discriminant capacity of lactoferrin to identify participants with cognitive impairment (PET+ versus PET-)

Area under the ROC curve	p-value	Criteriona	Sensibility (95%-CI)	Specificity (95%-CI)
0.667	0.034	<36.2	69.2 (42.8-85.7)	70.8 (48.9-87.4)

aCutt-off point given the highest Youden Index

## Data Availability

The datasets used and/or analyzed during the current study are available from the corresponding author.
